# Water pipe (shisha) smoking among male students of medical colleges in the eastern region of Saudi Arabia

**DOI:** 10.4103/0256-4947.62838

**Published:** 2010

**Authors:** Attia Z. Taha, Amr A. Sabra, Zaid Z. Al-Mustafa, Hasan R. Al-Awami, Mujtaba A. Al-Khalaf, Momen M. Al-Momen

**Affiliations:** From the Family and Community Medicine Department, King Faisal University, Dammam, Saudi Arabia; From the College of Medicine, King Faisal University, Dammam, Saudi Arabia

## Abstract

**BACKGROUND AND OBJECTIVES:**

Shisha smoking, one of the commonest methods of smoking tobacco among Arabs, increases the risk of cardiovascular and respiratory diseases. The objective of this study was to determine the prevalence of shisha smoking among male students of three colleges and to identify factors associated with shisha smoking.

**METHODS:**

This cross-sectional study included 500 male students of three colleges (medicine, applied medical sciences and dentistry). Data were collected from 371 students using a self-administered questionnaire.

**RESULTS:**

The overall prevalence of shisha smoking was 12.6% (8.6% shisha only and 4.0% both shisha and cigarettes). Thirty students (63.8%) started shisha smoking at ages of 16 to 18 years. Seven students (15%) smoked shisha daily. Cafés or restaurants were the favorite places for smoking (70.2% of students). There was a high prevalence of shisha smoking among students whose mothers had a secondary (19.1%) and higher (53.3%) education.

**CONCLUSIONS:**

There was a high prevalence of shisha smoking among university students. The majority of students started shisha smoking at a young age. Public health measures, including the banning of smoking in public places are recommended.

Shisha smoking (using a waterpipe, narghile or hookah) is a social and entertainment behavior of increasing popularity, especially among adolescents.^1^–^3^ Shisha smoke contains high concentrations of carbon monoxide, nicotine, tar and heavy metals.^4^,^5^ Thus, shisha smokers are at a greater risk of serious respiratory diseases and cancers.^6^–^9^ Shisha smoking is highly prevalent in developing countries and in the Eastern Mediterranean region.^10^–^12^

In Saudi Arabia there is a recent trend toward increased Shisha smoking.^13^–^16^ Saudi adolescents nowadays spend part of their leisure time smoking shisha in cafes and restaurants. Several studies have shown that shisha smoking is practiced more frequently (either daily or once per week).^17^,^18^ The favorite places for smoking shisha are with friends in café and open places.^16^,^17^,^19^ The objective of this study was to determine the prevalence of shisha smoking among male students from three medical colleges of King Faisal University in Dammam, Saudi Arabia and to identify factors associated with shisha smoking.

## METHODS

This was a cross-sectional study conducted at King Faisal University in Dammam city, Eastern Province, Saudi Arabia during the year 2008. The target population consisted of all male students (levels 1 to 3) of the three colleges namely, medicine, applied medical sciences and dentistry, with 572 students registered for the academic year 2008. Other levels (levels 4 to 6) were excluded because they usually study outside the university campus and were not accessible during the time of the study. The study was approved by the research committee of the College of Medicine and the university authorities. The objectives of the study were explained to the participating students after which they gave their informed consent. The information on individual students was kept confidential. The study sample was calculated by the computer package Epi Info (Epi Info TM version 3.3.2, 2005, CDC, Atlanta, USA) according to the total number of the students with an estimated average prevalence of shisha smoking of 10%^20^ at a confidence level of 95%. Thus, an estimated sample size of 500 students was included in the study using a simple random sampling technique. Three hundred seventy-one students responded (a response rate of 74.2%). They were distributed as follows: medicine (n=225 of 322 students), applied medical sciences (n=95 of 167 students), and dentistry (n=51 of 76 students. All Saudi and non-Saudi students were included in the study. A pilot study was conducted to test the validity and the logistics of the study. Subjects selected in the pilot study were excluded.

Data were collected through a self-administered questionnaire constructed by the investigators. It included questions on socio-demographic variables, current shisha smoking, smoking pattern and personal and family factors. Data collected were checked for accuracy and completeness, coded and entered into the SPSS 16 (SPSS Inc., Chicago, Illinois, USA) in a personal computer. Associations were determined by chi-squared test and statistical significance was taken as P<.05.

## RESULTS

The majority of students were from the College of Medicine (n=225, 60.6%) and were of Saudi nationality (n=367, 98.9%). Approximately half of the students' fathers had a higher education and higher family income. Two hundred sixty-six (72%) students lived with their families. The overall prevalence of shisha smoking was 12.6% (n=47), including shisha only (n=32) and both shisha and cigarettes (n=15). (Table 1). There was a high prevalence of cigarette smoking among fathers (n=51, 13.7%), and 5.4% (n=20) of the students' fathers as well as 2.4% (n=9) of mothers smoked shisha. Twenty-eight students (7.6%) had a shisha smoker in their residence. The majority of university students (n=30, 63.8%) started shisha smoking at the ages between 16 to 18 years, while 8 students (17.1%) started smoking before the age of 16 years (Table 2). Seven students (15%) smoked shisha daily, while 9 (19%) smoked 2 to 3 times per week. Cafés or restaurants were the favorite places for shisha smoking for the majority of students (n=33, 70.2%). Twenty-nine students (62%) spent 1 to 3 hours in shisha smoking per one session. A high proportion of students had the habit of smoking shisha with friends at any time (n=31; 66%), after eating (n=25; 53.2%) and with tea and coffee (n=18; 38.3%). About one-fifth of students smoked shisha during examination times (n=10, 21.3%) and when angry or stressed (n=9, 19.1%).

**Table 1:** Distribution of the university students (n=371) according to their smoking status.

Characteristics	No.	%
Smoking status		
Non-smokers	313	84.4
Shisha only	32	8.6
Cigarettes only	11	3.0
Both cigarettes and shisha	15	4.0
Father		
Non-smoker	297	80.1
Shisha	20	5.4
Cigarettes	51	13.7
Shisha and cigarettes	1	0.3
Refused to answer	2	0.5
Mother		
Non-smoker	358	96.5
Shisha	9	2.4
Cigarettes	1	0.3
Refused to answer	3	0.8
Presence of smokers in place of residence		
Non-smoker	258	69.6
Shisha	28	7.6
Cigarettes	61	16.4
Shisha and cigarettes	22	5.9
Refused to answer	2	0.5
Smoking wife among married students (n=8)		
Non-smoker	5	62.5
Shisha	1	12.5
Refused to answer	2	25.0

**Table 2:** Smoking habits of the students (n=47).

	No.	%
Age of starting shisha smoking		
10-12 years	2	4.3
13-15 years	6	12.8
16-18 years	30	63.8
≥18 years	3	6.4
Refused to answer	6	12.8
Frequency of shisha smoking Once per week	24	51.1
2-3 times per week	9	19.1
> 3 times per week	2	4.3
Daily	7	14.9
Refused to answer	5	10.6
Time spent in shisha smoking in one session		
<1 hour	13	27.7
1-3 hours	29	61.7
>3 hours	2	4.3
Refused to answer	3	6.4
Favorite places for shisha smoking^a^		
Café or restaurants	33	70.2
Open places	14	29.8
Home	3	6.4
Sports clubs	1	2.1
Any place with friends	20	42.6
Conditions associated with shisha smoking		
Tea and coffee drinking	18	38.3
After eating	25	53.2
When feeling happy	5	10.6
When angry or stress	9	19.1
During examination times	10	21.3
With friends at any time	31	66.0

a Response categories were not totally exclusive.

A significantly higher proportion of shisha smokers were students from the College of Medicine compared with the other two colleges (Table 3). More education among mothers was significantly associated with shisha smoking. Shisha smoking was practiced more in houses where there were no smokers in the residence.

**Table 3:** Variables associated with shisha smoking status of university students.

Variables	Shisha smokers (n=47)		Non-shisha smokers (n=324)		Total (n=371)		χ^2^-test (df)	*P*
	No.	%	No.	%	No.	%		
College								
Medicine	19	40.4	206	63.6	225	60.6	16.6 (2)	<.001
Applied Medical Sciences	13	27.7	82	25.3	95	25.6		
Dentistry	15	31.9	36	11.1	51	13.7		
Mother's education								
Illiterate or read and write	6	12.8	68	21.0	74	19.9	14.1 (5)	.015
Primary	1	2.1	40	12.3	41	11.1		
Intermediate	5	10.6	41	12.7	46	12.4		
Secondary or diploma	9	19.1	77	23.8	86	23.2		
University or higher education	25	53.2	91	28.1	116	31.3		
Refused to answer	1	2.1	7	2.2	8	2.2		
Presence of smokers in place of residence							
Yes	21	44.7	90	27.8	111	29.9	5.6 (1)	.018
No	26	55.3	234	72.2	260	70.1		

## DISCUSSION

The overall prevalence of shisha and cigarette smoking was 12.6% with a prevalence of 8.6% for shisha smoking only. This result was higher than that reported by Almutairi study of 7.3% among university students in Riyadh.^15^ It was also higher than the result shown by Abolfotouh et al in Abha (34 shisha smokers out of 489 students).^16^ The Al-Turki study conducted in central Saudi Arabia revealed that among medical student smokers, 44.1% smoked shisha and 23.7% smoked both cigarettes and shisha.^21^ A high prevalence of shisha smoking was also shown by other studies from the Gulf and Eastern Mediterranean countries.^11^,^17^,^19^,^22^ A survey among 937 British university students showed a prevalence of 8.0% for regular waterpipe smokers.^23^

The high prevalence of shisha smoking among King Faisal University students should raise the awareness of public health and administrative authorities about this problem. Approximately 64% of university students started shisha smoking at the ages 16 to 18 years. This result is consistent with the Maziak et al study in which the mean (SD) age of initiation of shisha smoking was 19.2 (2.2) years.^12^ It was also consistent with the Mohammed et al study in Kuwait where 30% started shisha smoking at ages 14 to 17 years.^24^ These findings show that shisha smoking is becoming more popular among adolescents. The harmful consequences of shisha smoking will soon hit the productive sector of the population. Adding to this, the finding of this study is that shisha smoking was practiced frequently (15% smoke shisha daily, 51% once per week and 19%, 2-3 times per week), which is in agreement with other studies. Other studies have shown that shisha smoking was practiced more frequently.^11^,^17^,^18^,^24^ Thus students are more exposed to the inhalation of toxic materials in tobacco. It is therefore a growing and unrecognized public health issue.

Cafés or restaurants were the favorite places for shisha smoking in our study. This result was similar to the Anjum et al^19^ and Abolfotouh^16^ et al. Several studies have shown that shisha smokers were significantly more likely to have shisha smokers as friends.^12^,^24^ These findings show the influence of friends and peer pressure on shisha smoking. The attitudes and beliefs of the parents, the availability of recreation facilities and the regulations governing smoking at public places should be considered when planning for intervention strategies.

The higher proportion of shisha smokers among students of the medical college might possibly reflect the higher socioeconomic status of their families. Anjum et al study showed that the highest percentage of shisha smoking was observed among college students in a higher socioeconomic group.^19^ The finding that a large proportion of shisha smokers have mothers with higher levels of education was interesting. One explanation is that a highly educated mother might consider shisha smoking prestigous and an indication of a modern standard of living. Our results are in agreement with several studies that showed a high prevalence of shisha and cigarette smoking among students of highly educated parents.^11^,^19^ Further studies are needed to explore the influence of parents' education on smoking. A high proportion of shisha smokers did not have smokers in their residency. The difference was statistically significant. This result was not in accordance with other studies which showed that smoking is associated with the presence of a smoker in the residence.^12^,^16^,^24^

A limitation of this study is the response rate (74.2%), which was lower than the calculated sample size. This might be due to the fact that some students preferred not to reveal that they were smokers. In conclusion, this study showed a high prevalence of shisha smoking among male university students with the majority starting smoking at a younger age. The study also revealed the influence of friends and peers on the initiation and continuation of shisha smoking. Public health interventions, including laws and regulations governing smoking at restaurants and cafes, should be implemented.
